# Differential proteomic analysis demonstrates follicle fluid participate immune reaction and protein translation in yak

**DOI:** 10.1186/s12917-021-03097-0

**Published:** 2022-01-14

**Authors:** Jie Pei, Rende Song, Pengjia Bao, Mancai Yin, Jiye Li, Guomo Zhang, Fude Wu, Zhengjie Luo, Xiaoyun Wu, Weiru Song, Yang Ba, Lin Xiong, Chunnian Liang, Xian Guo, Ping Yan

**Affiliations:** 1 Lanzhou Institute of Husbandry and Pharmaceutical Sciences, Chinese Academy of Agricultural Sciences, Lanzhou, China; 2 Key Lab of Yak Breeding Engineering in Gansu Province, Lanzhou, China; 3 Center of Yushu Tibetan Autonomous Prefecture for Animal Disease Prevention and Control, Yushu, China; 4 Datong Cattle Farm in Qinghai Province, Xining, China

**Keywords:** Yak, Ovarian follicle, Proteomics, iTRAQ, Immune reaction, Protein translation

## Abstract

**Background:**

Ovarian follicle fluid (FF) as a microenvironment surrounding oocyte plays critical roles in physio-biochemical processes of follicle development and oocyte maturation. It is hypothesized that proteins in yak FF participate in the physio-biochemical pathways. The primary aims of this study were to find differentially expressed proteins (DEPs) between mature and immature FF, and to elucidating functions of the mature and immature FF in yak.

**Results:**

The mature and immature FF samples were obtained from three healthy yaks that were nonpregnant, aged from four to five years, and free from any anatomical reproductive disorders. The FF samples were subjected to mass spectrometry with the isobaric tags for relative and absolute quantification (iTRAQ). The FF samples went through correlation analysis, principle component analysis, and expression pattern analysis based on quantification of the identified proteins. Four hundred sixty-three DEPs between mature and immature FF were identified. The DEPs between the mature and immature FF samples underwent gene ontology (GO), Kyoto encyclopedia of genes and genomes (KEGG), and protein-protein interaction (PPI) analysis. The DEPs highly expressed in the mature FF mainly took parts in the complement and coagulation cascades, defense response, acute-phase response, response to other organism pathways to avoid invasion of exogenous microorganisms. The complement activation pathway contains eight DEPs, namely C2, C5, C6, C7, C9, C4BPA, CFH, and MBL2. The three DEPs, CATHL4, CHGA, and PGLYRP1, take parts in defense response pathway to prevent invasion of exogenetic microorganism. The coagulation cascades pathway involves many coagulation factors, such as F7, F13A1, FGA, FGB, FGG, KLKB1, KNG1, MASP1, SERPINA1, and SERPIND1. While the DEPs highly expressed in the immature FF participated in protein translation, peptide biosynthetic process, DNA conformation change, and DNA geometric change pathways to facilitate follicle development. The translation pathway contains many ribosomal proteins, such as RPL3, RPL5, RPS3, RPS6, and other translation factors, such as EIF3J, EIF4G2, ETF1, MOV10, and NARS. The DNA conformation change and DNA geometric change involve nine DEPs, DDX1, G3BP1, HMGB1, HMGB2, HMGB3, MCM3, MCM5, MCM6, and RUVBL2. Furthermore, the expressed levels of the main DEPs, C2 and SERPIND1, were confirmed by western blot.

**Conclusions:**

The differential proteomics revealed the up-regulated DEPs in mature FF take parts in immunoreaction to prevent invasion of microorganisms and the up-regulated DEPs in immature FF participate in protein synthesis, which may improve our knowledge of the follicular microenvironment and its biological roles for reproductive processes in yak. The DEPs, C2 and SERPIND1, can be considered as protein markers for mature yak follicle.

**Supplementary Information:**

The online version contains supplementary material available at 10.1186/s12917-021-03097-0.

## Background

As a member of bovidae family, yak (Bos grunniens) is an only bovine breed that adapts to severe cold and low oxygen levels in high altitude regions ranging from 2,500 m to 5,500 m [1]. Over ninety percent of the global yak population distributes in plateau areas of China, which are characterized by their high elevations, pristine natural environments, and extreme seasonal variations [2]. Yak provide food, transport, shelter and fuel for nomads living in the plateau regions [3, 4]. Fecundity is a particularly important economic feature in yak production [5, 6]. Reproductive efficiency of female yak is determined by normal follicle development, oocyte maturation, and ovulation. However, as a highly seasonal breeding animal, yaks generally have a long interval from calving to the next conceiving. A minority of yaks return to estrus in the following breeding season, and most yaks begin the shift from a non-reproductive status to a reproductive status in the second, even in the third year [5].

During estrus, mammalian ovarian follicles commence dilating, follicular antra fill with follicle fluid (FF), granulosa cells initiate dividing, and Graafian follicles start to forming [7–10]. The follicle development is a precise and complex physiological process orchestrated by several biochemical factors [11]. The composition of FF results from secreted products from the mural and granulosa, thecal cells with minor contribution from the oocytes, and blood plasma constituents that cross blood follicular barrier via theca capillaries of theca layer [12]. The FF provides a very special microenvironment surrounding oocyte and contains various regulatory molecules, and hence, it plays critical roles in follicle development, oocytes maturation, fertilization success, and subsequent embryo quality [13]. The FF biochemical composition reflect not only the developmental state of the ovarian follicle, but also oocyte competence [14]. Therefore, understanding of the molecular characterization in the biological fluid could contribute to discover biomarkers with predictive values for fecundity. Proteins are the important composition of FF, which may take part in regulation of the follicle development and oocyte maturation. Thus, investigation of contents and changes of FF in protein expression may give us valuable information on which protein mediators lead to follicle development, and which proteins can be considered as biomarkers of oocyte quality.

Within the past several years, proteomics has been widely applied to the identification and quantification of proteins from complex biological samples [15–19], including FF samples from a variety of species [20–24]. However, there are still limited studies on the protein composition of FF in hope of shedding light on specific reproductive mechanisms of intricate protein networks and pathways in yak. Previously, differential proteins between mature and immature yak FF were detected by two-dimensional gel electrophoresis (2-DE) to find the regulatory factors controlling generation of dominant follicle [25]. However, only a few proteins with remarkably highly expressed levels were identified in the yak FF, owing to technical resolution limitations of the 2-DE. To our knowledge, there are no studies that have used mass spectrometry-based proteomic technology to compare protein components of mature FF with that of immature FF in preovulatory phase of yak.

The primary aim of this study was to search for protein composition differences between mature and immature yak FF by the leading-edge proteomic method, in order to identify proteins that may hold a key to the reproductive process and further may be identified as potential biomarkers for follicle/oocyte quality. In the present study, the yak FF in mature and immature statuses were used as research material, and differentially expressed proteins (DEPs) between the two types of the yak FF were identified and quantified by isobaric tags for relative and absolute quantification (iTRAQ). The expressed levels of main DEPs were detected by western blot technology to confirm the quantifications obtained by iTRAQ technology. The DEPs went through a wide variety of bioinformatic analyses in combination with the most up-to-date public databases to understand the representative biological functions and pathways in which the DEPs play the vital roles. The results of the present study will be beneficial to finding the regulatory factors of the ovarian FF maturing, explaining the reproductive mechanisms of the complex ovulatory process, discovering new methods of improving reproductive efficiency for yak.

## Results

### Enrichment of low-abundant proteins

To avoid potential interference from highly abundant proteins, a compression of the dynamic range of FF protein concentration was conducted by a sample preparation tool. The results of enrichment for the FF proteins were monitored using SDS-PAGE of the crude, flow-throughs, and bound fractions, and their protein contents were determined by the BCA assay. The enrichment treatment compressed the dynamic range of protein concentrations, reduced the concentrations of high-abundance proteins, and increased the concentrations of low- and medium-abundance proteins. The compression for FF samples removed around 90% of total loaded protein amount.

### Proteomic changes between mature and immature follicle fluid

The enriched samples were subjected to iTRAQ analysis to find DEPs between mature and immature FF. The mass spectrometry proteomics data have been deposited to the ProteomeXchange Consortium (http://proteomecentral.proteomexchange.org) via the iProX partner repository [26] with the dataset identifier PXD025426. A total of 1955 proteins were identified in the yak FF, whose comprehensive information about the identified proteins is available as supplementary material to this paper (Additional file 1). Proteins with fold changes greater than 1.2 and p-values less than 0.05 were considered as DEPs between mature and immature FF. A total of 463 DEPs were identified through above screening criteria. Out of the 463 DEPs, 140 proteins and 323 proteins were up-regulated and down-regulated respectively in the mature FF, compared with those in the immature FF. Comprehensive information of the DEPs are presented in Additional files 2 and 3.

### Expression pattern analyses on proteins in mature and immature follicle fluid

**Fig. 1:**
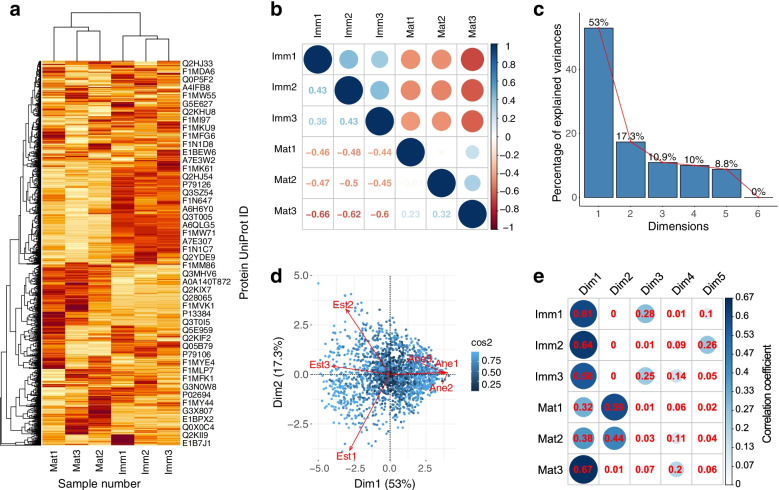
Heatmap, correlation analysis, and principal component analysis (PCA) for the follicle fluid (FF) samples based on quantification of the all identified proteins in yak. **a** Heatmap shows the global expression difference of the FF samples based on the total identified proteins. **b** Correlation matrix illustrates correlations among the yak FF samples of the mature and immature FF. **c** Scree plot exhibits the first six principal component of the PCA for the FF samples. **d** PCA biplot shows the first two principal components (Dim1 and Dim2). The correlation of each variable with each axis is indicated by the length and angle of lime segments. **e** Correlation matrix illustrates correlations between each yak FF sample and each principal component of the first five principal components.

Many bioinformatic analyses were applied to explore underlying functions of the DEPs. Through the heatmap of the total identified proteins (Fig. 1a), the yak FF samples were gathered into the two groups as grouped by the follicle sizes, indicating the two highly clustered sampled-based within-group expression patterns. When analyzed in the correlation matrix with all the FF samples, there are positive correlation coefficients between each two intragroup samples, as well as negative correlation coefficients between each two intergroup samples (Fig. 1b).

The screeplot displays the proportion of the total variation explained by the five principal components, and the first two components cumulatively explain approximately 70% of the variance for the protein expression data (Fig. 1c). The biplot graph based on PCA was constructed to illustrate the compositional similarities/differences among the FF samples. In the two-dimension biplot, the mature FF group and immature FF group were opposite to each other, which indicates their highly negative correlation (Fig. 1d). The correlations between each FF sample and each principal component were calculated using Spearman correlation and visualized using the R package “corrplot”. The first two principal components (Dim1 and Dim2) demonstrate higher positive correlation coefficients with the FF samples (Fig. 1e).

**Fig. 2:**
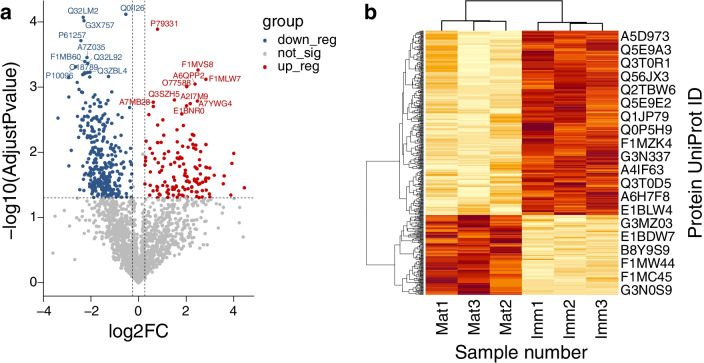
Volcano plot and heatmap indicating the significantly differentially expressed proteins (DEPs) in yak follicle fluid (FF) samples. **a** Volcano plot illustrates the DEPs in yak FF samples. The up-regulated proteins are shown in red and the down-regulated proteins are shown in blue. The PDB ID are given to the top 10 up- and down- regulated proteins. **b** Heatmap exhibits the DEPs in the yak FF samples

The volcano plot for the total proteins in yak FF demonstrate the up- and down-regulated DEPs in the mature FF group compared with immature FF group (Fig. 2a). The expression profiling changes of the DEPs in the yak FF was visualized according to their relative expressed levels in the heatmap (Fig. 2b). There are more down-regulated DEPs than up-regulated DEPs in the mature FF samples displayed in the two graphs.

### Pathway enrichment for the differential expressed proteins

**Fig. 3:**
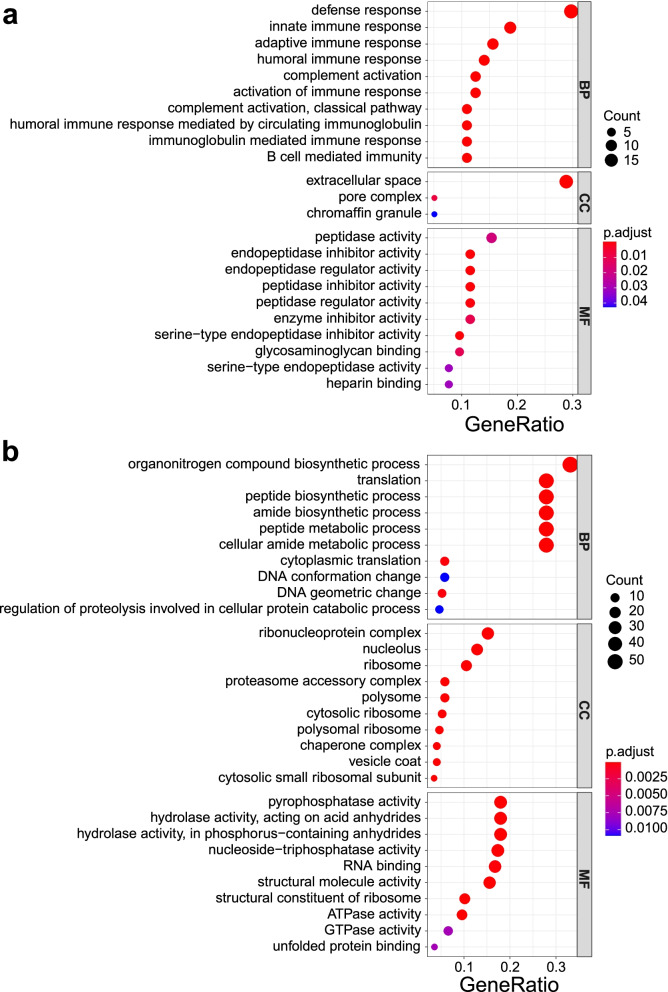
Bubble diagrams of Gene Ontology (GO) enrichment analysis for the Differentially expressed proteins (DEPs) between mature and immature yak follicle fluid (FF). **a** Bubble diagram of the top 10 GO BP pathways for the up-regulated DEPs demonstrates the proteins are primarily implicated into defense response, innate immune response, adaptive immune response, humoral immune response, and complement activation. **b** Bubble diagram of the top 10 GO BP pathways for the down-regulated DEPs indicates the proteins are mainly involved in organonitrogen compound biosynthetic process, translation, peptide biosynthetic process, amide biosynthetic process, and peptide metabolic process

The bubble plot represents the top 10 enriched pathways for the up-regulated DEPs of the mature FF group using GO as a reference database (Fig. 3a). The functional characterization of the up-regulated proteins in the mature FF shows that the enriched proteins associated with GO-BP categories mainly involved in defense response, immune response, complement activation, acute-phase response, cell killing, and defense response to fugus (Additional file 4). The complement activation pathway mainly contains C2, C5, C6, C7, C9, C4BPA, CFH, and MBL2, which are responsible for complement cascade reaction. Three DEPs, CATHL4, CHGA, and PGLYRP1, participate defense response, defense response to fugus, and cell killing. The top 10 enriched pathways in GO-BP terms of the up-regulated DEPs of the immature FF group are shown in another bubble plot (Fig. 3b). GO analysis reveals the enriched pathways for the up-regulated DEPs of the immature FF involved in protein translation, peptide biosynthetic process, peptide metabolic process, DNA conformation change, posttranscriptional regulation of gene expression, protein localization, protein catabolic process, and nucleoside metabolic process (Additional file 5). The translation pathway contains 48 DEPs including 38 ribosomal proteins, such as RPL3, RPL5, RPS3, RPS6, and other translation factors, such as EIF3J, EIF4G2, ETF1, MOV10, and NARS. The 48 proteins also take parts in organonitrogen compound biosynthetic process, peptide biosynthetic process, amide biosynthetic process, peptide metabolic process, and cellular amide metabolic process. Nine DEPs participate DNA conformation change and DNA geometric change, including DDX1, G3BP1, HMGB1, HMGB2, HMGB3, MCM3, MCM5, MCM6, and RUVBL2.

**Fig. 4:**
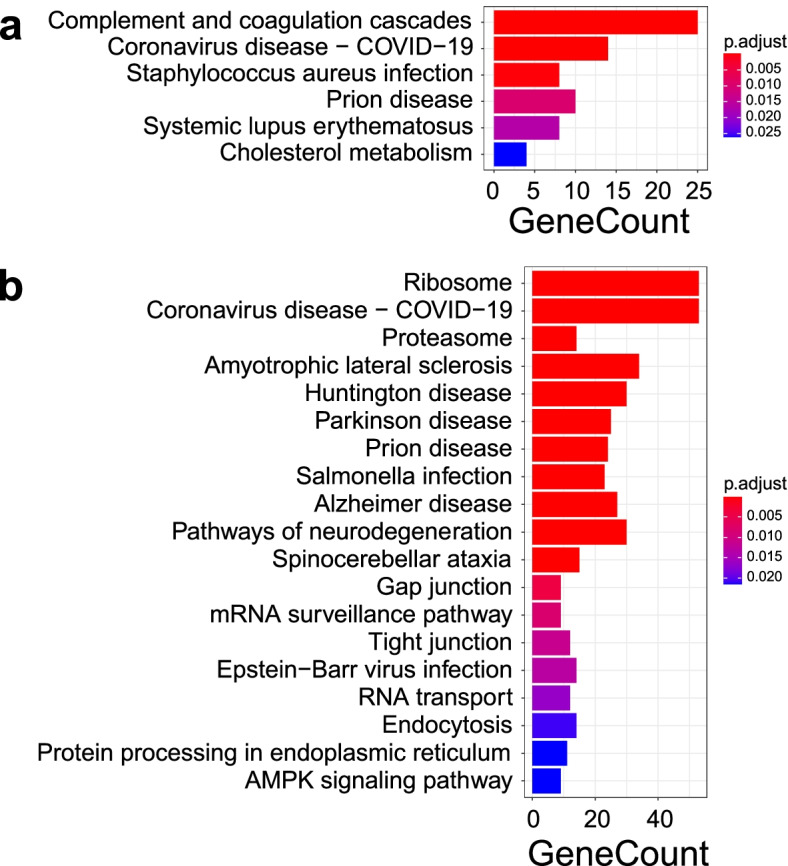
Barplots of the enriched pathways of Kyoto Encyclopedia of Genes and Genomes (KEGG) pathway enrichment analysis for the Differentially expressed proteins (DEPs) between mature and immature yak follicle fluid (FF). **a** Barplot of the six KEGG pathways for the up-regulated DEPs in the mature FF. **b** Barplot of the nineteen top KEGG pathways for the down-regulated DEPs in the mature FF

The barplot of KEGG pathway analysis for the up-regulated DEPs in the mature FF indicates that 6 KEGG pathways with four or more genes were significantly enriched, including complement and coagulation cascades, coronavirus disease, staphylococcus aureus infection, prion disease, systemic lupus erythematosus, and cholesterol metabolism (Fig. 4a). In line with the enriched GO-BP pathways, the complement and coagulation cascades also contain C2, C5, C6, C7, C9, C4BPA, CFH, and MBL2. Besides these complement family members, this pathway involves many coagulation factors, such as F7, F13A1, FGA, FGB, FGG, KLKB1, KNG1, MASP1, SERPINA1, and SERPIND1. In the barplot of KEGG pathway analysis for the up-regulated DEPs in the immature FF, 19 KEGG pathways with nine or more genes were significantly enriched, mainly including ribosome, coronavirus disease, proteasome, amyotrophic lateral sclerosis, Huntington disease, Parkinson disease, prion disease, and salmonella infection (Fig. 4b). Compared with the enriched GO-BP pathways, the ribosome pathway comprises 53 ribosomal proteins, containing the 38 ribosomal proteins from GO-BP categories. The detailed information of the KEGG pathways were showed in Additional files 6 and 7.

### Protein-protein interaction among the differential expressed proteins

**Fig. 5:**
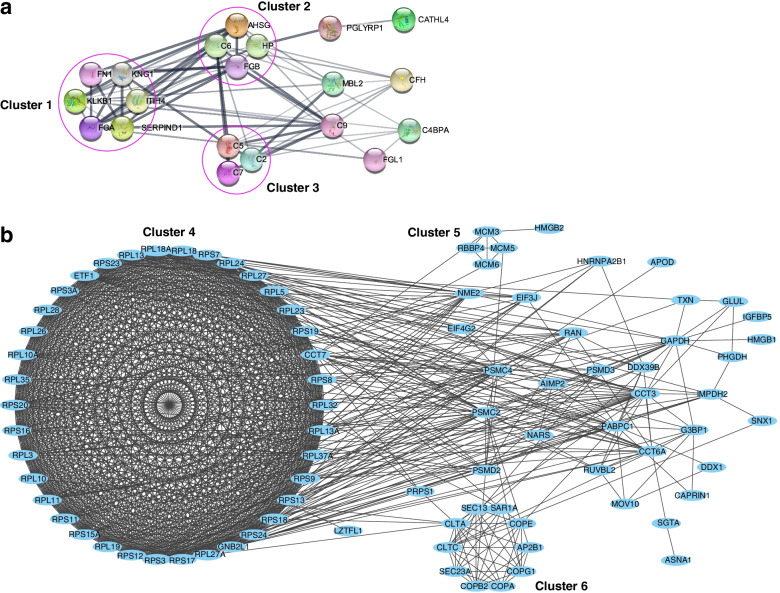
Networks of the protein-protein interaction (PPI) of the differentially expressed proteins (DEPs) enriched by Gene Ontology (GO) enrichment analysis and subsequently screened by Molecular Complex Detection (MCODE) plugin of Cytoscape. The functional modules of the PPI network were shown in the clusters. **a** Network of the PPI for the up-regulated DEPs in the mature FF. The cluster 1 takes parts in defense response, acute-phase response, and acute inflammatory response. The cluster 2 participates in defense response, acute-phase response, response to other organism, and acute inflammatory response, and response to biotic stimulus. The cluster 3 plays roles in complement activation. **b** Network of the PPI for down-regulated DEPs in the mature FF. The cluster 4 participates in translation, peptide biosynthetic process, peptide metabolic process, and amide biosynthetic process. The cluster 5 takes parts in DNA geometric change, DNA conformation change, and DNA duplex unwinding. The cluster 6 performs its functions in Golgi vesicle transport, cellular protein localization, and cellular macromolecule location

The networks of PPI were established respectively for the up-regulated DEPs in mature and immature yak FF, which were selected by GO enrichment analysis. Through filtered by MCODE, the functional modules of the PPI networks were obtained respectively for the up-regulated protein groups in mature and immature FF. For the up-regulated DEPs in the mature FF, there are obviously three main grouped functional clusters extracted from the PPI network (Fig. 5a), which are mainly responsible for defense response (Cluster 1, 2), acute-phase response (Cluster 1, 2), response to other organism (Cluster 2), response to biotic stimulus (Cluster 2), and complement activation (Cluster 3). At the same time, the three major clusters (Cluster 4~6) were generated from the PPI network of the up-regulated DEPs in the immature FF (Fig. 5b). Cluster 4 mainly contains numerous ribosomal protein components that participate protein translation, peptide biosynthetic process, and peptide metabolic process. There are four filtered proteins in Cluster 5 that take part in DNA geometric change, DNA conformation change, and DNA duplex unwinding. Cluster 6 comprises 10 filtered proteins that participate in many cellular functions including Golgi vesicle transport, protein localization, and macromolecule localization.

### Immunoblotting verification of protein changes typical for yak follicle fluid

**Fig 6:**
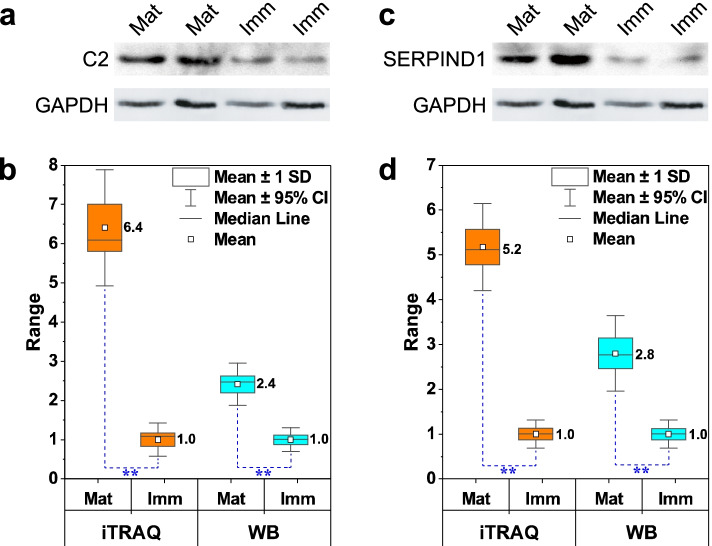
Western blot (WB) analysis for C2 and SERPIND1 in the yak follicle fluid (FF) samples. **a** Representative WB bands for target protein C2 and internal reference protein GAPDH. **b** Boxplot shows the relative expression levels of C2 detected by iTRAQ and WB respectively. The asterisks stand for extremely significant difference (*p*<0.01). **c** Representative WB bands for target protein SERPIND1 and internal reference protein GAPDH. **d** Boxplot indicates the relative expression level of SERPIND1 detected by iTRAQ and WB respectively. The asterisks stand for extremely significant difference (*p*<0.01)

To further validate the results from the quantitative proteomic analysis, the identity of the DEPs between mature and immature yak FF was confirmed by the immunoblot experiments. The two key proteins named as C2 and SERPIND1 were selected to verified the relative quantification of the proteomic analyses. The yak FF samples were separated by SDS-PAGE and transferred onto PVDF membranes followed by immunodetection using specific antibodies. The results shown in Fig. 6 confirmed significantly higher levels of C2 and SERPIND1 in mature versus immature FF.

## Discussion

As a seasonal breeding mammal, yak is sexually inactive during the non-breeding season, which severely restricts its population reproductive capacity [5]. This phenomenon may be caused by developmental restriction of follicles and oocytes during anestrus. It is essential to understand the molecular mechanism of yak breeding phenotype, and further to provide some prospective information for improving its fecundity. The present study was designed to identify new candidate proteins potentially influencing follicle development and maturation in FF and to explore underlying functions of the follicle in the different phases determining reproduction processes. The understanding of the precise biochemical pathways affecting the final outcome of the reproductive process was of paramount value.

FF serves as a crucial microenvironment for both ovarian somatic growth and germ cell maturation [27]. It comprises substances involved in cell differentiation, gamete quality and follicle rupture [28]. The study depicting protein composition of yak will conductive to understanding the regulations of ovarian physiology and follicular development. The developmental state of ovarian follicle reflects mature state of oocyte contained in animal FF [29]. Leading follicle is most likely to possess a mature oocyte with increased fertilization and high quality embryo development capacity [30]. FF of mammals is derived from plasma and secretions of granulosa and theca cells [31]. Bidirectional communication between granulosa and oocyte is a fundamental part for growth and development of both follicle and oocyte [32]. As a significant component, proteins in FF plays crucial messenger roles in many important cellular functions and biological processes, including follicle growth and development, angiogenesis stimulation, oocyte maturation, ovulation, and fertility potential in a granulosa–oocyte information [33]. It is hypothesized that protein composition in FF can serve as potential biomarkers of follicle maturation and oocyte competence in yak, which is in line with the research for assessment oocyte quality in human [24]. So, protein components in yak FF was chosen as a study object to research mechanism for follicle and oocyte development.

Good quality oocytes are exceedingly critical for success of in vitro fertilization (IVF) and subsequent derivation of high-quality embryos. During estrus, the oocyte matures in the milieu of FF in which signaling molecules circulate between granulosa and oocyte [34]. Follicular growth is monitored by transrectal ultrasonography for animals undergoing in vitro fertilization (IVF) [35–37]. There is a lack of markers to assess oocyte quality and to predict success of the IVF treatment for yak. Up to now, signal biomolecules, such as gonadal hormones, gonadotropins, gonadotrophin releasing hormones, inhibiting factors, and cytokines, have not been verified as reliable markers of oocyte maturation [38, 39]. The proteins in dominant FF were mainly implicated to the advanced states of follicle maturation, prior to subsequent ovulation. With current lack of diagnostic markers, the yak FF represents a rich pool of proteins useful as a source of prognostic and/or diagnostic biomarkers. Application of omics technologies, such as proteomics, may be extremely conductive not only to discovering relevant group of candidate biomarkers, but additionally to understanding complex regulatory networks involved in ovarian physiology and oocyte maturing in yak.

In the present study, the comparative proteomic study indicated that the up-regulated DEPs in the mature FF were principally enriched in the GO-BP pathways related to defense response, immune response, and complement activation (Fig. 3a). In fact, all the identified DEPs associated with the immunoregulation functions in mature FF had higher expression levels than those in immature FF. For KEGG analysis, the up-regulated proteins mainly take parts in complement and coagulation cascades, coronavirus disease, and staphylococcus aureus infection (Fig. 4a).

Complement system is a cornerstone of innate immunity for organisms. It is composed of over 30 proteins and is activated in response to invading pathogens, allogenic materials, tissue injury, apoptosis, and necrosis [40]. The complement system includes three main complement activation pathways, the classical pathway, the lectin pathway, and the alternative pathway [41]. In the present study, eight up-regulated proteins (C2, C5, C6, C7, C9, C4BPA, CFH, and MBL2) were clustered into a large group of complement proteins participating the complement cascade (Fig. 5a). Consistent with our findings, many previous studies reported that the members in complement system were characterized as important constituents of FF and played essential roles in reproductive processes [42–47]. The highly expressed levels of these complement factors are a strong proof that the mature FF take part in complement cascade activation.

The higher levels of the complement components C2, C5, C6, C7, C9, and C4BPA together in the mature yak FF observed in this study clearly indicated the distinctive regulation of complement cascade in the yak FF. The higher level of complement factor H was observed in the mature FF, which may be allowing the alternative pathway to remain inactive before ovulation. It seems that controlling complement activity in follicle fluid before ovulating may contribute to normal oocyte maturation. Complement regulatory proteins played an important role in prevention of harmful amplification of the complement cascade [48]. Additionally, the complement activation resulted in insufficient of free vascular endothelial growth factor (VEGF), that could capture the angiogenic factor required for normal placental development in high soluble level [49]. Restrained complement activation prevented these angiogenesis failure and rescued pregnancies [50]. Moreover, the inhibition of the complement system is required for maintaining oocyte viability [51].

Interestingly, the complement system, traditionally delineated as a linear cascade of separate pathways, is currently considered a hub-like network that carry out a global mediator in immune surveillance, cell and tissue homeostasis and repair which is tightly connected to other systems [52]. Several researches indicate that ovulation can be considered as an inflammatory event involving the innate immune system [53], also based on the presence of a large amount of acute-phase response proteins in FF [53–55]. The previous results support a role for the complement system in the associated inflammatory response and suggest that this role is of relevance in folliculogenesis [56]. The complement activation is important in inflammation and may facilitate the physiological and local inflammatory reaction of ovulation [57]. In this regard, our results demonstrated that several DEPs, up-regulated in mature FF versus immature FF, were clustered in the acute phase response signaling pathway, such as AHSG, ITIH4, FN1, and HP (Fig. 5a). It can be deduced that the dramatic increase in the up-regulation of these proteins in the follicle compartment activates the ovarian innate immune system. The phenomenon may be deciphered by the result that complement activation can also initiate inflammation via recruitment and activation of inflammatory cells [58]. Furthermore, the high levels of acute phase proteins in mature yak FF highlighted a possible involvement of inflammatory process related to wound healing. The process resembles wounding and wound healing, and the genes involved in ovulation strongly resemble those orchestrating a sterile inflammatory reaction [53, 59]. Collectively, this endorses the hypothesis that an inflammatory-like process is involved in rupture, release of the oocyte.

In a previous study, the functional enrichment analysis showed a particular balance of functions related to the acute phase response signaling and defense response [56]. Complemental system targets infectious microbes for destruction, clears immune complexes, and apoptotic cells from the circulation, and augments the humoral response. In the present study, the GO enrichment indicated that the up-regulated proteins in the mature FF played essential roles in response to other organisms and response to fungus. In agreement with previous reports in the porcine FF proteomic results [60], the up-regulated protein in the mature FF enriched in Staphylococcus aureus infection pathway by the KEGG analysis. The path way include C2, C5, CATHL4, CFH, FGG, MASP1, MBL2, and PLG. All these findings strongly suggest yak require more antimicrobial proteins against exogenous organisms in its mature FF during ovulation.

In the present study, the enrichment analyses resulting from both GO and KEGG indicated that major up-regulated proteins in the immature FF are associated with protein translation, particularly with the structure and function of ribosome. The translation pathway contains 48 DEPs including 38 proteins composing ribosomal structural components, such as RPL3, RPL5, RPS3, RPS6, and other translation factors, such as EIF3J, EIF4G2, ETF1, MOV10, and NARS. The enriched ribosome pathway includes 53 ribosomal proteins containing the above 38 ribosomal components, confirming the high reliability of the enrichment results. Interestingly, nearly forty proteins with ribosome structural components were significantly higher expressed in ovaries of Dorset sheep with low prolificacy than in those of Small Tail Han sheep with high prolificacy [61]. These results imply that the presence of a large number of the ribosome structural proteins is a distinct feature of the immature follicle. Moreover, immature FF had a higher expression level of the proteins that participate DNA conformation change and DNA geometric change, including DDX1, G3BP1, HMGB1, HMGB2, HMGB3, MCM3, MCM5, MCM6, and RUVBL2, suggesting there were more transcriptional activities for the protein synthesis in the immature FF. Furthermore, we found several new proteins in yak FF but their possible roles in reproduction have not been investigated.

Although the present study contributes to the field of yak reproduction, it still has some limitations that need to be taken into account. The yak FF has a high dynamic range of protein concentrations, which attenuates the ability of the proteomic technology to detect low abundant proteins and their changes. The relatively low number of individual samples per group may have caused incorrect conclusions if the selected yaks were not representative of the population. Prospective studies with a larger sample size are expected to confirm of the findings of the current study. Furthermore, the differential protein expressions were evaluated between the mature and immature FF to identify DEPs potentially associated with the oocyte quality. It remains to be determined whether these proteins identified in the FF contribute directly to follicular development and oocyte quality in yak FF, or are merely biomarkers for them. For more authentic results, further studies are required on extended sample size and different sample cohorts to more accurately understand the function of DEPs in FF.

## Conclusion

Many DEPs between mature and immature FF were identified and quantified by the iTRAQ technology in yak. These proteins together with their regulatory pathways may play vital roles in reproductive processes. The high level of immunoregulatory factors in the mature FF may regulate immune system to defense exogenous microorganisms during yak ovulation, the high level of ribosomal protein in the immature FF may improve translation intensity to prepare for material needs of ovulation in yak immature FF. Simultaneously, a set of key proteins as potential biomarker candidates were discovered to identify mature ovarian follicles and oocytes. The proteins participating complement and coagulation cascades, like C2, C5, C6, C7, C9, C4BPA, CFH, MBL2, F7, F13A1, FGA, FGB, FGG, KLKB1, KNG1, MASP1, SERPINA1, and SERPIND1, can be considered as protein markers for mature yak follicle. The proteins comprising ribosome and participating DNA conformation change, such as RPL3, RPL5, RPS3, RPS6, DDX1, G3BP1, HMGB1, HMGB2, HMGB3, MCM3, MCM5, MCM6, and RUVBL2, can be regarded as protein markers for immature yak follicle.

## Methods

### Follicle fluid collection

Due to the difficulty of obtaining yak FF through intravital follicular aspiration, most studies on the mechanisms of ovulation and luteal transition has been performed by means of yak slaughter test. Healthy female yaks who live in more than 3200 meters above sea level were recruited at a local abattoir in Xining City, Qinghai Province, China. The yaks aged from 4 to 5 years were nonpregnant, free from any anatomical reproductive disorders. Yaks devoid of any active corpus luteum on their both ovaries, with their biggest follicles above 10 mm in diameter, was selected for FF aspirating. The ovaries were removed immediately from the yak carcasses after slaughter. Concentrations of estradiol and progesterone in each follicle were determined to decide the status of the follicle. The follicle was regarded as in a preovulatory phase and selected for further experiments when the estradiol/progesterone ratio was above 1. The biggest follicle in diameter from 10 to 13 mm on bilateral ovaries was considered as mature follicle, and smaller follicles in diameter from 3 to 5 mm were regarded as immature follicles.

Each yak FF sample was directly aspirated from puncture of the ovarian follicle by a 1-mL sterile syringe with a 20-guage needle. Then, the yak FF was transferred to a 2-mL microfuge tube. The mature FF in the dominant follicle was transferred into one tube, and all the immature FF was combined into another tube for each yak. Only clear FF samples, indicating lack of macroscopic blood contamination, were considered in the study. The FF was immediately centrifuged at 500 g, at 4 °C for 10 min in order to remove cells and insoluble particles. The supernatant was transferred to a new 2-mL microfuge tube and centrifuged a second time at 12,000 g, at 4 °C for 30 min to remove cellular components and debris. Then, the fluid supernatant was transferred to a new sterile polypropylene tube and frozen in liquid nitrogen until further analyses. Yak ovarian FF from 6 yaks were used for the further proteomic analysis and western blot.

### Sample preparation

The yak FF samples were suspended in protein Lysis buffer (7 M Urea, 2 M Thiourea, 4% CHAPS, 40 mM Tris-HCl (pH 8.5), 1 mM PMSF, and 2 mM EDTA) and then sonicated in ice. The proteins were reduced with 10 mM DTT (final concentration) at 56 °C for 1 h and alkylated by 55 mM IAM (final concentration) in the darkness at room temperature for 1 h. The reduced and alkylated protein mixtures were precipitated by adding 4× volume of chilled acetone at -20 °C overnight. After centrifugated at 4 °C, 30,000g, the pellet was dissolved in 0.5 M TEAB (Applied Biosystems, Milan, Italy) and sonicated in ice. After centrifuged at 30,000 g at 4 °C, protein concentration of the supernatant was determined by BCA Protein Assay kit (Thermo Scientific, Rockford, IL). Aliquoted protein solutions were kept at -80 °C for further analyses.

### Low-abundance protein enrichment

Presence of highly abundant proteins in the FF samples can adversely impact upon the ability to identify and quantify low-abundance proteins for proteomic analysis. To overcome limitations of commonly used proteomic technology related to a wide dynamic range of protein concentrations in the FF, which makes it hard to identify low-abundance proteins, the yak FF were treated by a sample preparation tool used for compression of the dynamic range of protein concentration. A ProteoMiner Kit (BioRad Laboratories, Inc.) without species-specificity compared with the immune-affinity method, which employs highly diverse bead-based library of combinatorial peptide baits [62], was applied to enriching low-abundance proteins. The enrichment of yak FF samples was carried out according to the manufacturer’s protocol with a few modifications. Briefly, the yak FF samples were clarified by centrifugation at 10,000 g for 10 min at 4 °C. Two hundred microliter of the sample was added to a column and the column was rotated on a rotational shaker for 2 h at room temperature. After incubated, the column was centrifuged at 1000 g for 45 sec. The bound column was washed with 200 μL of wash buffer by centrifugation at 1000 g for 45 sec three times. The bound material was eluted with 20 μL aliquots of four different elution buffers in the same centrifugation condition as described above. Fractions of the crude, flow-throughs, and eluates were detected by sodium dodecyl sulphate polyacrylamide gel (SDS-PAGE) electrophoresis for evaluation of enrichment efficiency. Total protein concentration for each eluate fraction was determined by BCA assay (Thermo Fisher Scientific).

### iTRAQ labeling and SCX fractionation

Total protein (100 μg) in each yak FF sample solution was taken and then digested with Trypsin Gold (Promega, Madison, WI, USA) with the ratio of protein : trypsin =30 : 1 at 37 °C for 16 h. After trypsin digestion, peptides were dried via vacuum centrifugation. The peptides were reconstituted in 0.5 M triethyl ammonium bicarbonate (TEAB) and processed according to the manufacture’s protocol for 8-plex iTRAQ reagent (Applied Biosystems). In brief, one unit of iTRAQ reagent was thawed and reconstituted in 24 μL isopropanol. Samples were labeled with iTRAQ tags as follow: the digested peptide samples from three immature FF specimens were labeled with iTRAQ tags 113, 114, 116, and the digested peptide samples from three mature FF specimens were labeled with iTRAQ tags 117, 119, 121. The peptides were incubated at room temperature for 2 h for labelling with the isobaric tags. The labeled peptide mixtures were pooled and then dried by vacuum centrifugation.

Strong cation exchanger (SCX) chromatography was performed with a LC-20AB HPLC Pump system (Shimadzu, Kyoto, Japan). In details, the iTRAQ labeled peptide mixtures were reconstituted with 4 mL buffer A (25 mM NaH2PO4 in 25% ACN, pH 2.7) and loaded onto a 4.6×250 mm Ultremex SCX column containing 5 μm particles (Phenomenex). The peptides were eluted at a flow rate of 1 mL/min with a gradient of buffer A for 10 min, 5-60% buffer B (25mM NaH2PO4, 1 M KCl in 25% ACN, pH 2.7) for 27 min, and 60-100% buffer B for 1 min. The system was then maintained at 100% buffer B for 1 min and equilibrated with buffer A for 10 min prior to the next injection. The peptide elution was monitored by absorbance measurements at 214 nm, and the eluted fractions were collected at 1-min intervals and divided into 10 fractions. Each fraction was desalted with a Strata X C18 column (Phenomenex) and then vacuum-dried.

### LC-ESI-MS/MS analysis based on Triple TOF 5600

Each fraction was resuspended in buffer A (5% ACN, 0.1%FA) and centrifuged at 20,000 g for 10 min. The final concentration of each peptide was approximately 0.5 μg/μL on average. Ten microliters of supernatant were loaded onto a 2-cm C18 trap column by an autosampler in an LC-20AD nano HPLC (Shimadzu, Kyoto, Japan). Then, the peptides were eluted onto a 10-cm analytical C18 column (inner diameter 75 μm) packed in-house. The samples were loaded at 8 μL/min for 4 min, and the 35 min gradient was run at 300 nL/min starting from 2% to 35% buffer B (95%ACN, 0.1%FA), followed by a linear gradient to 60% buffer B for 5 min, a linear gradient to 80% buffer B for 2 min, a maintenance at 80% buffer B for 4 min, and a finally linear gradient to 5% buffer B in for 1 min.

Data acquisition was performed with a TripleTOF 5600 System (AB SCIEX, Concord, ON) fitted with a Nanospray III source (AB SCIEX, Concord, ON) and a pulled quartz tip as the emitter (New Objectives, Woburn, MA). Data was acquired using an ion spray voltage of 2.5 kV, curtain gas of 30 psi, nebulizer gas of 15 psi, and an interface heater temperature of 150 °C. The MS was operated with a reverse power of greater than or equal to 30,000 FWHM for TOF MS scans. For IDA, survey scans were acquired in 250 ms and as many as 30 product ion scans were collected if exceeding a threshold of 120 counts per second (counts/s) and with a 2+ to 5+ charge-state. The total cycle time was fixed to 3.3 s, and the Q2 transmission window was 100 Da for 100%. Four time bins were summed for each scan at a pulse frequency value of 11 kHz through monitoring of the 40 GHz multichannel TDC detector with four-anode channel detect ion. A sweeping collision energy setting of 35±5 eV coupled with iTRAQ adjust rolling collision energy was applied to all precursor ions for collision-induced dissociation. Dynamic exclusion was set for 1/2 of peak width (15 s), and then the precursor was refreshed off the exclusion list.

### Protein identification

Raw data (.wiff files) acquired from the Orbitrap Triple TOF 5600 System were converted into peak lists (.mgf files) (5600 MS converter) using Proteome Discoverer 1.2 (Thermo Fisher Scientific, Massachusetts, US). Protein identification and quantification based on the recorded MS/MS spectra were carried out by using ProteinPilot 4.5 software (ABSciex) against a bovine database containing 46,728 sequences downloaded from Uniprot database. All common fixed and variable modifications of proteins were considered. iTRAQ 8 plex was selected for sample type. The enzyme specificity was selected as “trypsin”. Mass tolerance for initial peptides mass was selected as 20 ppm, and mass tolerance for fragmented ions was set to 0.1 Da, with allowance for one missed cleavage in the trypsin digests. Cysteine alkylation modification was selected as MMTS. The corresponding reversed database was adopted to evaluate the false discovery rate (FDR) for peptide identification, which were estimated from the target-decoy search approach. The peptide for quantification was automatically selected by the Pro Group algorithm to calculate the reporter peak area, error factor, and p-value.

### Screening for differentially expressed proteins

Statistical differences of DEPs were determined using R software (version 4.0.3). Student's t-test were used, and a p-value < 0.05 was considered to indicate a statistically significant difference. Each confident protein identification involves at least one unique peptide. For protein quantitation, it was required that a protein contains at least two unique peptides. Fold change for each protein was calculated as the quotient of the average values obtained respectively from the two sample groups. Protein with a fold change above 1.2 and a p-value below 0.05 between the two groups was considered to be DEP.

### Bioinformatics analyses for differentially expressed proteins

Hierarchical clustering and heatmap visualization of all the identified proteins was performed by the R package “stats” according to hierarchical clustering on the basis of the expression matrix. Correlation analysis of all the identified proteins was executed by the R package “corrplot” (version 0.84) based on Spearman correlation coefficients, and the plot was made by the R package “ggplot2”. Screeplot, biplot, and correlation plot were generated using the R package “FactoMineR” to conduct Hierarchical agglomerative clustering, “factoextra” to plot PCA screeplot and biplot, and “corrplot” to made correlation plot.

The R packages “ggpubr” and “ggthemes” were applied to construct volcano plot of the DEPs between the mature and immature FF. Heatmap of the DEGs was plotted by the R package “stats” as above described.

Gene ontology (GO) and Kyoto encyclopedia of genes and genomes (KEGG) terms of the DEPs were enriched using the R package “clusterProfiler”, “GO.db”, and “org.Bt.eg.db” to identify significant biological process (BP), cellular component (CC), and molecular function (MF). The bubble diagrams for the enrichments were then created by the R package “ggplot2” to explore the GO pathways for up-regulated DEPs in mature and immature FF respectively. Then, the bar diagrams of KEGG pathway analysis for the DEPs were plotted by the R package “ggplot2”.

The protein-protein interaction (PPI) networks for DEPs enriched respectively from the up-regulated protein groups of the mature and immature FF by GO analysis were constructed and displayed by STRING plugin of Cytoscape software to validate the enrichment results, and to understand the potential regulatory mechanisms. The functional modules of the PPI network were subsequently screened by Molecular Complex Detection (MCODE) plugin of Cytoscape.

### Immunoblot and quantitative analysis

Aliquots of the total protein extracts enriched from the yak FF (10 μg) were separated in 12% SDS-PAGE gels using Mini-PROTEAN Tetra Cell System (Bio-Rad). The proteins were transferred onto a polyvinylidene difluoride membranes by eBlot protein transfer system (Genscript, Piscataway, NJ) in a semi-dry transfer buffer (50 mM Tris, 190 mM glycine, 0.1% SDS, 20% methanol). The membrane was blocked in Tris-buffered saline-Tween (pH 7.4) containing 5% skimmed milk at room temperature for 2 h, and incubated with primary antibodies raised against GAPDH (Abcam Inc., ab181603, 1:1000), C2 (Abcam Inc., ab209900; 1:1000) and SERPIND1 (Origene Inc., TA332957, 1:1000) at 4 °C overnight. After a series of washes, the membrane was subsequently incubated with secondary anti-rabbit IgG antibodies conjugated with horseradish peroxidase (ZSGB-BIO Inc., TA08, 1:2000) in TBST containing 5% skimmed milk at room temperature for 1 h. Finally, immunodetection signal was detected with an ECL chemiluminiscence reagent (GE Healthcare), and visualized with ChemiDoc XRS system (Bio-Rad, Hercules, CA). Protein bands of each sample were quantified by densitometry with ImageJ software. Relative expression levels of the target proteins were normalized by calculating from ratios of the gray levels of their bands against those of the corresponding GAPDH bands. The expression level values of C2 and SERPIND1 were extracted from the expression matrix of iTRAQ quantification. The expression differences between mature and immature FF were visualized by boxplots constructed by the Origin software version 2019b, based on the relative expressed levels of the two proteins respectively from the iTRAQ and WB quantification.

## Supplementary Information

**Additional file 1.** All identified proteins.

**Additional file 2.** Upregulated DEPs in mature FF.

**Additional file 3.** Upregulated DEPs in immature FF.

**Additional file 4.** GO of upregulated DEPs in mature FF.

**Additional file 5.** GO of upregulated DEPs in immature FF.

**Additional file 6.** KEGG of upregulated DEPs in mature FF.

**Additional file 7.** KEGG of upregulated DEPs in immature FF.

**Additional file 8.** WB digital images.

## Data Availability

The mass spectrometry proteomics data generated during the current study are available in the ProteomeXchange Consortium via the iProX partner repository, [http://proteomecentral.proteomexchange.org/cgi/GetDataset?ID=PXD025426]. Other raw datasets may also be requested from the corresponding author provided that all ethical requirements have been met.

## Abbreviations

FF: Follicle fluid
DEP: Differentially expressed protein
iTRAQ: Isobaric tags for relative and absolute quantification
GO: Gene ontology
KEGG: Kyoto encyclopedia of genes and genomes
PPI: Protein-protein interaction
2-DE: Two-dimensional gel electrophoresis
IVF: In vitro fertilization
VEGF: Vascular endothelial growth factor
SDS-PAGE: Sodium dodecyl sulphate polyacrylamide gel
TEAB: Triethyl ammonium bicarbonate
SCX: Strong cation exchanger
FDR: False discovery rate
BP: Biological process
CC: Cellular component
MF: Molecular function
MCODE: Molecular complex detection
