# Supplementary material for: Differential proteomic analysis demonstrates follicle fluid participate immune reaction and protein translation in yak
Source: BMC Vet Res. 2022 Jan 14;18:34. doi: 10.1186/s12917-021-03097-0 (PMC8758897; doi:10.1186/s12917-021-03097-0)
Supplement: Supplementary file 8 — Additional file 8. WB digital images. [file 12917_2021_3097_MOESM8_ESM.pdf]

Title page for WB images

Differential proteomic analysis demonstrates follicle fluid participate immune reaction and protein translation in yak

Jie Pei[1,4], Rende Song[2], Pengjia Bao[1,4], Mancai Yin[3], Jiye Li[3], Guomo Zhang[3], Fude Wu[3], Zhengjie Luo[3], Xiaoyun Wu[1,4], Weiru Song[2], Yang Ba[2], Lin Xiong[1,4], Chunnian Liang[1,4], Xian Guo[1,4]*, Ping Yan[1,4]*

[1]Lanzhou Institute of Husbandry and Pharmaceutical Sciences, Chinese Academy of Agricultural Sciences, Lanzhou, China
[2]Center of Yushu Tibetan Autonomous Prefecture for Animal Disease Prevention and Control, Yushu, China
[3]Datong Cattle Farm in Qinghai Province, Xining, China
[4]Key Lab of Yak Breeding Engineering in Gansu Province, Lanzhou, China

The full-length Western Blot images of GAPDH, C2, and SERPIND1 are exhibited in the next page. The cropped blot images in the manuscript are shown in red frames.

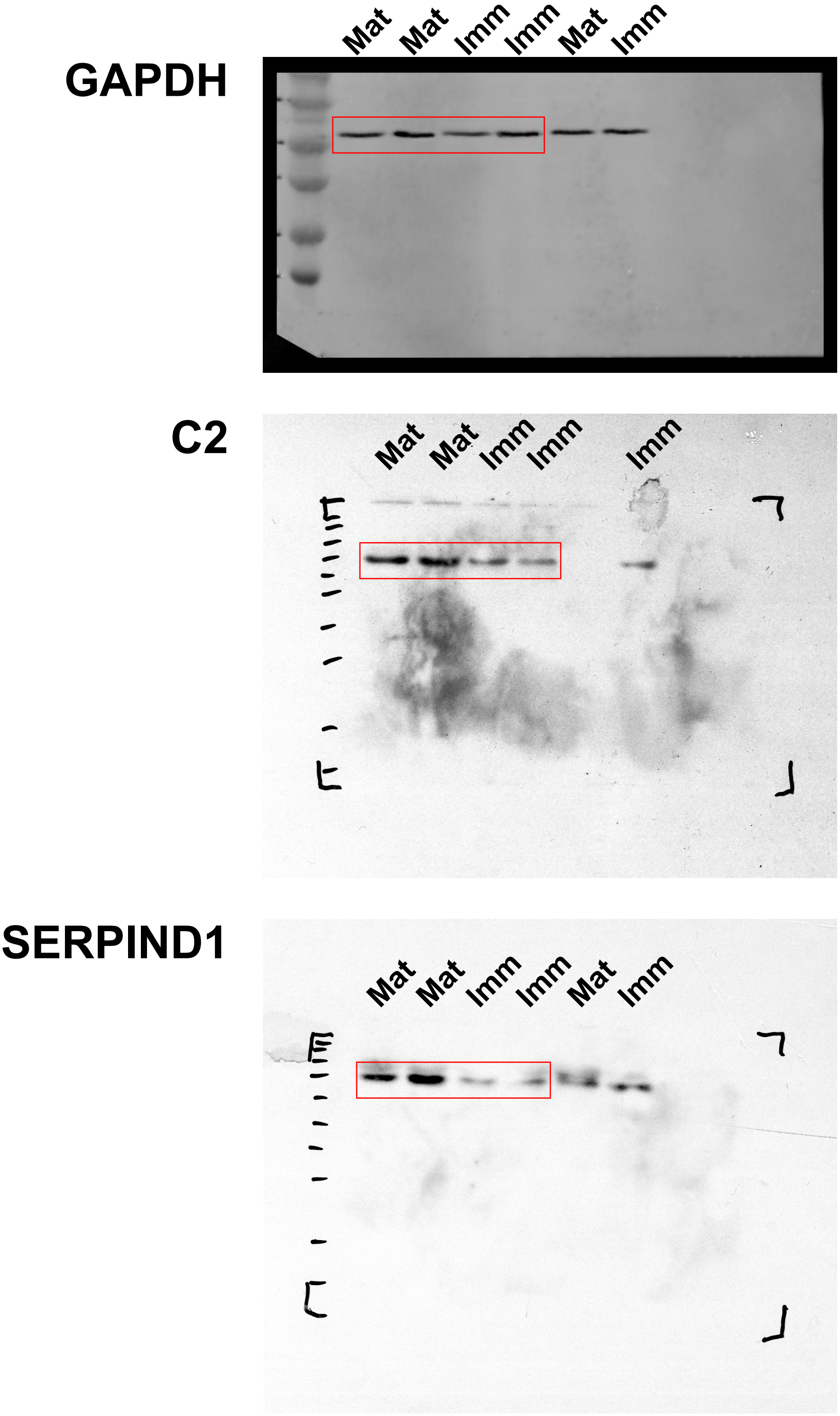
GAPDH
Mat
Mat
Imm
Imm
Mat
Imm
C2
Mat
Mat
Imm
Imm
Imm
SERPIND1
Mat
Mat
Imm
Imm
Mat
Imm
